# Network analysis of school absence: central symptoms and their functions

**DOI:** 10.3389/frcha.2025.1625164

**Published:** 2025-11-20

**Authors:** Katarina Alanko, David Heyne, Martin Lagerström, Martin Knollman

**Affiliations:** 1 Centre for Learning Analytics, University of Turku, Turku, Finland; 2 Department of Humanities, The Faculty of Human and Social Sciences, Åbo Akademi University, Turku, Finland; 3 School of Psychology, Faculty of Health, Deakin University, Burwood, VIC, Australia; 4 Department for Child and Adolescent Psychiatry, Psychosomatics, and Psychotherapy, LVR Clinic Dusseldorf, Heinrich-Heine University Dusseldorf, Dusseldorf, Germany

**Keywords:** school absence, network analysis, adolescence, symptoms, functions, inventory of school attendance problems

## Abstract

**Objective:**

School attendance problems (SAPs) often develop and persist through complex interactions among numerous influences. This study used network analysis to identify: (i) the most central symptoms reported by youths with SAPs; (ii) the most central functions underlying those symptoms; and (iii) the relationships among symptoms and among functions.

**Method:**

Self-reported symptoms and their functions were assessed via an online survey comprising the Inventory of School Attendance Problems. We analysed data from Finnish middle school students (*M* age = 14.9 years, range 12–17, gender: 40% male, 57% female, 3% other) reporting school absence of ≥10%. There were 349 responses for symptoms and 333 responses for functions. Network analysis was used to identify the most central symptoms and functions (nodes) along with the associations between different symptoms, and the associations between different functions (edges).

**Results:**

Results indicated complex networks among the symptoms and among the functions. Depression emerged as the descriptively most central node in both networks. In the symptoms network, it showed moderate links to Performance Anxiety, Aggression, and Social Anxiety. In the functions network, its strongest links were to School Aversion/Attractive Alternatives and Social Anxiety. Social Anxiety was also highly central in the functions network, with links to Agoraphobia/Panic and Problems with Peers. All 13 nodes were interlinked in both the symptoms and functions networks, reflecting widespread co-occurrence among symptoms and among functions.

**Conclusions:**

Depression's central position across both the symptom and function networks may make it a valuable intervention target, even when other symptoms are also salient.

## 1 Introduction

For some youth, school is not experienced as a supportive place to learn and grow, but rather as a source of distress that can contribute to persistent absence (1). Such difficulties are often captured under the umbrella of “school attendance problems” (SAPs), a collective term for the range of difficulties youth can experience when it comes to going to school or staying there (2, 3). SAPs develop over time, and occur on a dimension of problem severity, ranging from school reluctance, in the form of verbally expressing distress about going to or being at school, through occasional absences, to long periods of continued absence (4). A 10% threshold is used in many countries as a cut-off for describing absence as problematic (e.g., (5–7). Short- and long-term consequences of problematic absence from school include academic problems, social isolation, mental health problems, and difficulty completing secondary education (2, 3).

There is substantial heterogeneity in the reasons for SAPs and the ways they present (2, 8). Reflecting this heterogeneity, SAPs have been studied not only collectively, but also according to specific types. Heyne et al. (2) outlined four main types, namely school refusal (hereafter referred to as emotion-related absence), truancy, school withdrawal, and school exclusion. Emotion-related absence (i.e., when a young person is reluctant or refuses to attend school because of emotional distress) occurs among 1%–7% of youth in the general population and 5%–16% of youth in clinical settings (9–11). Truancy (i.e., being absent without the permission of parents or school) occurs among 20% of middle school youth on an occasional basis, and among 4% more frequently (12). The prevalence of school withdrawal (i.e., when a parent directly or indirectly supports absence) and school exclusion (when a school directly or indirectly supports absence) is less well-established (13, 14). However, a recent study indicated that 3.9% of middle school students reported school withdrawal, and 1.7% reported school exclusion (15). Absence from school due to illness is common, occurring among 22% (12) to 35% (16) of middle school students during the prior month. Illness absences may stem from common subjective health complaints such as headache and nausea, and may be related to anxiety (10). Overall, the prevalence of SAPs appears to be rising (6, 7, 12), especially after the COVID-19 pandemic (17, 18). Given this, and the detrimental impact of SAPs on young people, interventions to prevent and reduce SAPs are urgently needed.

Comprehensive assessment is central to understanding SAPs and planning intervention (19). Symptoms are often co-occurring, making it difficult to identify the most relevant targets for intervention (20). For example, Ingul and Nordahl (21) examined the impact of various psychosocial factors on school attendance by comparing high school students with and without SAPs, but with identical levels of school anxiety. They found that problems such as fewer having friends at school, being bullied, and not being treated with respect distinguished anxious non-attenders from equally anxious attenders. Also, bullying is more strongly associated with emotion-related absence than truancy in primary schools, while social isolation was more frequent among lower-secondary students with emotion-related absence (10). Another example is the common co-occurrence of depression and anxiety (22), which not only frequently overlap but also impact areas such as social interaction and physiological processes, including sleep and appetite (23–25). Indeed, just as particular depressive symptoms may contribute to the development of other depressive symptoms (23), symptoms associated with SAPs may contribute to the development of other SAP-related symptoms. Furthermore, just as alleviating a depressive symptom may reduce the severity of other depressive symptoms (26), we propose that alleviating a symptom associated with SAPs could help reduce not only related symptoms, but possibly the SAP itself. These patterns highlight the importance of identifying core symptoms that may be central to the prevention and amelioration of SAPs.

Expanding beyond symptom identification, researchers have also sought to understand the “functions” of SAPs. First, Kearney and colleagues (27, 28) distinguished between functions related to negative reinforcement and those related to positive reinforcement of school absence. Negative reinforcement includes the avoidance of negative affect that would otherwise be experienced at school, and the avoidance of social and/or evaluative situations at school, such as feeling embarrassed in front of others. Positive reinforcement of absence includes receiving attention from significant adults when staying at home, and gaining access to more satisfying activities outside of school. Kearney's functional model is operationalized in the School Refusal Assessment Scale (and its revised versions), a widely used questionnaire that focuses on the functions of behavior in young people with established SAPs (28, 29).

Second, Knollmann and colleagues (30) conceptualize function—also referred to as impact—as equivalent to the reason for the SAP. This conceptualization informed the development of the Inventory of School Attendance Problems (ISAP), which explores both symptoms and their functions in parallel. Although symptom-function correlations are generally high (30, 31), it is theoretically possible that they may differ. For instance, a young person may report symptoms of depression, yet identify their primary reason for non-attendance as a desire to stay home with a parent. Furthermore, just as multiple SAP-related symptoms can co-occur, multiple functions may co-occur as well.

The ISAP is a relatively recent assessment tool, notable for two reasons. First, it enables the assessment of a broad range of factors potentially associated with SAPs, spanning individual, family, social, and school domains. Second, it allows for the simultaneous assessment of both the symptoms potentially associated with SAPs and the function each symptom may—or may not—serve in contributing to school absence. The relevant subscales are referred to as ISAP-S (symptoms) and ISAP-F (functions).

Based on research with a sample of German youth aged 8–19 years with school absence, recruited from outpatient child and adolescent psychiatry/psychotherapy clinics (i.e., specialist-referred), Knollmann et al. identified 13 factors across the 48 items of the ISAP by combining scores from the ISAP-S and ISAP-F scales. These factors are: Depression, Social Anxiety, Separation Anxiety, Performance Anxiety, Agoraphobia/Panic, Somatic Complaints, School Aversion/Attractive Alternatives, Aggression, Problems with Peers, Problems with Teachers, Dislike of the Specific School, Problems Within the Family, and Problems with Parents. The psychometric properties of the ISAP have since been examined in two studies using community samples, with support for the 13-factor model found through confirmatory factor analysis in both the Finnish and Swedish translations (32, 33).

Given the frequent co-occurrence of symptoms and their respective functions, a thorough investigation to their interrelations is warranted. However, even with detailed assessment, it may be difficult to identify core symptoms and functions that should be prioritized in intervention planning. In the initial study on the ISAP, Knollmann et al. (30) reported high correlations between most scales (e.g., Depression and Social Anxiety: *r* = .56; Aggression and Depression: *r* = .44; Problems with Peers and Social Phobia: *r* = .57). This pattern is consistent with findings from the School Refusal Assessment Scale—Revised, which is based on Kearney's functional model [e.g., Negative Affectivity and Social/Evaluative Fears: *r* = .61; cf (31)]. These findings, based on clinical samples, underscore the need to clarify the interrelations among symptoms and functions associated with SAPs. In addition, empirical knowledge is needed to identify core symptoms with a high functional impact—those that might serve as key intervention targets—both in clinical samples and in non-referred samples displaying early signs of SAPs.

Network analysis offers an innovative method for examining nuanced relationships at the item or factor level*.* It serves as an alternative to the latent variable approach used in traditional confirmatory factor analysis. In network analysis, direct associations (“edges”) amongst items or factors (“nodes”) are thought to constitute the latent construct itself (34). These factors, and their internal relationships, are conceptualized as active components that contribute to the development and maintenance of a disorder or phenomenon (35). Unlike traditional models, network analysis does not require pathways to be specified *a priori*. Instead, all potential pathways are estimated while accounting for the influence of all other variables in the model, after which the network is regularized to produce a clear and interpretable visual representation. Since many potential network models are estimated, regularization is used as a model-selection technique to overcome the problem of multiple comparisons by punishing more complex network models (i.e., models with a higher number of edges) that are hard to interpret (36). The most commonly used regularization technique for network models is the least absolute shrinkage and selection operator (LASSO); it detects edges that are weak and thus likely spurious (i.e., false positives), removing them from the network and rendering a sparser and more interpretable network model (36).

Central symptoms within a network are those that are most strongly connected to other symptoms (34). The absence of an edge between two variables indicates conditional independence—that is, any shared variance is accounted for by other variables in the network.

In sum, the ISAP provides a comprehensive framework for assessing SAPs. It captures both the symptoms experienced by young people and the functions those symptoms may serve in relation to school absence, across individual, family, social, and school domains. This provides a unique opportunity to study the interrelationships among symptoms and functions, and to identify those that may be most central to SAPs. Such knowledge has the potential to inform intervention efforts for young people with more concerning levels of absence—the focus of the current study—by highlighting symptoms or functions that could be prioritized in support planning.

The present study addressed two exploratory research questions using network analysis. First, when controlling for the complexity of factors associated with SAPs, are some symptoms more central than others? Second, are some functions more central than others? To ensure a clear focus on problematic absence—specifically SAPs, rather than occasional absences or reluctance to attend school despite regular attendance—the study was conducted with youth reporting ≥10% absence from school.

## 2 Method

### 2.1 Participants, sampling, and process

The current study drew upon data from the “School Absence in Finland” project.

Youth were recruited via 15 Finnish schools agreeing to participate in the project. Schools were recruited via numerous channels: (1) the webpages and email lists of organizations for teachers and principals; (2) teacher organizations' social media channels; and (3) various Facebook groups for school professionals. All channels shared information about the study and invited school personnel to participate in an online seminar in which the project was presented. The online seminar was conducted in January 2021, comprising presentations about school absence and information about the project. The 15 participating schools were located in southern and western Finland. Participating schools emailed parents with information about the project and requested consent for their child's participation. The parents of youth below 15 years were asked to complete an informed consent for their child's participation in the study. In addition, youth of all ages provided informed written consent at the time of data gathering.

Survey data were collected from youth during school time in May 2021. A research assistant or teacher was present in class during data collection to answer any questions and provide technical assistance if needed. To obtain data from persistently absent youth, school personnel engaged in individual outreach—contacting absent youth directly, visiting their homes if necessary, and inviting them to complete the questionnaire. The responses of 42 youth were collected in this way.

Data were initially collected from 2,137 youth, through both school-based administration of the questionnaire and individual outreach. Of these, 271 cases (12.7%) were excluded principally due to lack of consent, implausible age, or excessive missing data. From the remaining 1,866 respondents, we selected a subsample of 370 youth (19.8%) who self-reported school absence of ≥10% over the past 12 school weeks. This subsample was inspected for patterns of missing data, which were imputed where possible (see Data Analysis section for details). In the present analyses, we focused on youth with ≥10% school absence. In an earlier study using the full sample, where “days absent” was included as a separate node, the overall network structure was highly similar (32). In the current models, we did not include “days absent” as a node, because the questionnaire items were explicitly framed in relation to school absence, making its inclusion redundant. Importantly, the similarity of the network structures across the full and high-absence samples suggests that the associations identified are highly similar. An additional item in the ISAP asked youth to estimate how many days they had been absent from school during the past 12 school weeks. The researchers did not ask participants to distinguish between authorized and unauthorized absences. Response options were: 1 = Not at all, 2 = Sometimes (up to 4 school days missed), 3 = Often (5–12 school days missed), 4 = Very often (13–36 school days absent), 5 = Most of the time (37–48 days missed), and 6 = (Almost Always (more than 48 days missed). Responses ≥3 were selected. The included options correspond to absence ≥8.3% of the previous 12 weeks (where 12 weeks corresponds to 60 school days).

The average age of participants in the final sample was 14.91 years (*SD* = 0.84, range 12–17). Participant gender was male (40%), female (57%), or other (3%). According to the adolescents, their parent's highest educational level was university or higher education (51%), upper secondary school or vocational studies (25%), comprehensive school or equivalent (4%), with the rest of the youth either not knowing their parents' educational level or indicating none of the above (20%). Adolescents lived with both parents (76%), interchangeably with two parents (14%), with one parent (10%), or in residential childcare (under 1%).

The study was approved by the research ethics committee of Åbo Akademi University.

### 2.2 Measure

To measure symptoms and their function in relation to absence, participants were administered the Inventory of School Attendance Problems [ISAP; (30)]. This 48-item questionnaire was constructed as a screening tool for identifying problems linked to emerging and existing SAPs. The ISAP contains both a symptom scale (ISAP-S) and a function scale (ISAP-F). For each item (e.g., “Before or at school, I feel down or depressed”; “Before or at school, I'm afraid of exams”), the respondent is asked to report: (a) the extent to which the item applies to them (i.e., how true it is for them or how often it occurs); and (b) the extent to which it is why they miss school or find it hard to attend school (i.e., how true it is for them or how often this is the reason). Both questions are answered on a 4-point Likert scale (from “not true at all”/“never” to “very much true”/“very often”). Responses to part (a) of each item are used to form the symptom factors (ISAP-S), and responses to part (b) of each item are used to form the function factors (ISAP-F).

The 48 items of the ISAP reflect 13 factors. The items associated with each factor can be found in Appendix 1, and the questionnaire can be found at INSA.network. The 13 factors relate to the following subscales (reliability estimates in the current sample for each symptom subscale reported in parentheses): (1) Depression (*α* = .89, *ω_t_* = .95), (2) Social Anxiety (*α* = .85, *ω_t_* = .88), (3) Separation Anxiety (*α* = .82, *ω_t_* = .86), (4) Performance Anxiety (*α* = .85, *ω_t_* = .85), (5) Agoraphobia/Panic (*α* = .82, *ω_t_* = .87), (6) Somatic Complaints (*α* = .71, *ω_t_* = .81), (7) School Aversion/Attractive Alternatives (*α* = .77, *ω_t_* = .81), (8) Aggression (*α* = .82, *ω_t_* = .83), (9) Problems with Peers (*α* = .81, *ω_t_* = .86), (10) Problems with Teachers (*α* = .77, *ω_t_* = .78), (11) Dislike of the Specific School (*α* = .86, *ω_t_* = .86), (12) Problems Within the Family (*α* = .90, *ω_t_* = .90), and (13) Problems with Parents (*α* = .84, *ω_t_* = .86).

The internal consistency of the subscales was deemed to be adequate based on administration with the original sample [.75 ≤ *α* ≤ .88, 3 testlets/scale; (30)] and the current sample (.71 ≤ *α* ≤ .90,.72 ≤ *ω_t_* ≤ .95).

### 2.3 Data analysis

#### 2.3.1 Descriptive statistics

Descriptive statistics were used to summarise the characteristics of the sample and the key variables of interest.

#### 2.3.2 Handling missing ISAP data

For the ISAP-S, responses on the 48 items were incomplete for 56 of the 370 youth. Thirty-five of these 56 youth (62.5%) had less than 30% missing data. The pattern of missing data was inspected and handled using the *mice* (37) and *VIM* (38) packages, via which data was determined to be missing at random. Thus, missing values were multivariate imputed. For the ISAP-F, responses were incomplete on the 48 items for 80 of the 370 participants and missing values for 43 of these 80 youth (53.8%) could be multivariate imputed. Missing values were imputed using predictive mean matching to create complete data sets for the two network analyses. After imputation, the sample sizes were *N* = 349 for ISAP-S and *N* = 333 for ISAP-F.

#### 2.3.3 Conducting network analysis

All data preparation and analyses were performed in R version 4.2.0. The following R packages were used for data analysis and plotting: *qgraph* (39), *bootnet* (36), *mgm* (40), and *tidyverse* (41).

Mixed graphical models were estimated for both networks using the Extended Bayesian Information Criterion [EBIC; (42)] for the regularization tuning. The algorithm creates multiple competing network models, by estimating all potential pathways between variables and then either shrinking weak edges or setting them to zero according to tuning parameters. The model that is selected is the one that minimizes the EBIC and most accurately corresponds to the level of sparsity that is preferred in the network. Here, the tuning hyperparameter lambda controlling for sparsity was set to 0.25, following Haslbeck and Waldorp's (40) recommendation for creating a final, readily interpretable network model. In our network model, solid edges indicate positive associations, whereas dashed edges indicate negative associations. Moreover, the thickness of the line indicates the magnitude of the edge. Edges were estimated using nodewise regression, with each node both regressing and being regressed by all other nodes in the network. Edges thus represent partial correlations after combining the two regression coefficients two variables share (40). Additionally, as an effect size network models, upper-bound nodewise predictability (*R*^2^) can be calculated for each node by predicting the node from all other nodes in the network. The *R*^2^ obtained in this manner thus represents an upper-bound of how much variance in the node is maximally explained by the other variables. If the explained variance is low, it indicates that important variables may have been omitted from the model (43) and that future studies should try to incorporate additional variables to increase explained variance. In the present study, predictability was calculated for all nodes, indicated by the grey border around each node. The centrality index strength, which is the sum of the absolute weights of the edge connecting the node to all other nodes, was calculated to highlight which symptoms were most influential in the network (26, 44). We opted to only calculate strength centrality based on work by Bringmann and colleagues, who suggested that strength centrality is the most appropriate measure of centrality compared to other centrality measures such as degree and betweenness centrality used in other types of networks (45). Another popular centrality measure is expected influence, where instead of taking the absolute sum of edge weights, the raw sum of edge weights is calculated (46). In the case where all edges share the same sign (i.e., are positive or negative), strength centrality and expected influence will be identical. We tested for statistically significant differences in strength centrality using bootstrapping as suggested by Epskamp and colleagues (36). To assess redundancy in the network, that is, whether two nodes share too similar edges with the other nodes, the goldbricker algorithm with the Hittner method from the *networktools* package was used (47). Furthermore, in accordance with Epskamp et al. (36), we assessed the stability of the networks using case-dropping bootstrap to calculate the correlation stability (CS)-coefficient for both edges and centrality that indicates the proportion of the sample that can be excluded while still sharing a strong correlation (.70) with the original data. High stability indicates that the results are likely to replicate in future research, and the CS-coefficient should ideally be above 0.5 to indicate high stability.

### 2.4 Transparency

Materials and analysis code for this study are available upon request.

## 3 Results

### 3.1 Descriptives

The large majority of respondents described themselves as being absent frequently: 80% selected “often,” 15% “very often,” 3% “mostly,” and 2% “always.”

For each of the 13 ISAP-S scales and 13 ISAP-F scales, sum score variables were calculated for the items within a given scale, and these sum score variables were used in the network model. Means and standard deviations for each sum score variable are displayed in Table 1. Visual inspection indicates that the mean values for the symptom scales were consistently higher than the mean values for the equivalent function scales. For example, the mean symptom score for the six items related to Depression was higher than the mean function score for the same six items. Across all 13 symptom scales, the scale with the highest mean score was Depression. The same is true for the 13 function scales.

**Table 1:** Means and standard deviations, and responses ≥1.5 for the sum scores of the ISAP-S scales and the ISAP-F scales.

Variable (number of items)	ISAP symptom				ISAP function			
	*M*	*SD*	Range	*N* (%) symptoms ≥1.5^a^	*M*	*SD*	Range	*N* (%) function ≥1.5^a^
Depression (6)	5.58	4.68	0–18	87 (25)	3.33	3.95	0–18	37 (11)
Social anxiety (5)	3.42	3.66	0–15	52 (15)	1.68	2.76	0–15	22 (7)
Separation anxiety (4)	1.61	2.30	0–12	29 (8)	0.62	1.67	0–12	13 (4)
Performance anxiety (3)	2.67	2.56	0–9	75 (21)	1.14	1.97	0–9	28 (8)
Agoraphobia/Panic (4)	1.39	2.33	0–12	22 (6)	0.78	1.88	0–12	11 (3)
Somatic Complaints (3)	2.60	1.99	0–9	55 (16)	2.15	2.03	0–9	48 (14)
School Aversion/Attractive Alternatives (4)	5.09	3.18	0–12	145 (42)	2.31	2.81	0–12	48 (14)
Aggression (3)	2.76	2.52	0–9	73 (21)	0.89	1.78	0–9	19 (6)
Problems with Peers (4)	2.01	2.74	0–12	48 (14)	1.20	2.22	0–12	19 (6)
Problems with Teachers (3)	1.68	2.06	0–9	37 (11)	0.82	1.56	0–9	14 (4)
Dislike of the Specific School (3)	1.87	2.39	0–9	43 (12)	0.80	1.68	0–9	17 (5)
Problems Within the Family (3)	1.29	2.11	0–9	32 (9)	0.60	1.52	0–9	13 (4)
Problems with Parents (3)	1.06	1.88	0–9	22 (6)	0.45	1.34	0–9	9 (3)

a Mean symptom scores ranged from 0 to 3.

The cut-off value of a mean score of 1.5, indicating elevated scores, was chosen based on previous research (30).

To obtain a clear understanding of elevated scores in the subscales, we also report descriptive statistics of the proportion of participants who scored higher than a cut-off value of 1.5 on the ISAP subscales, consistent with the definition employed for descriptive purposes (i.e., mean scale value above or equal to half of the maximum of 3) in the original study conducted by Knollmann et al. (30). Of the youth who scored ≥1.5 on any of the subscales, symptoms of School Aversion (42%) and Depression (25%) were the most common, whereas functions of School Aversion and Somatic Complaints (both reported by 14%), followed by Depression (11%) were the most common (see Table 1).

### 3.2 Network estimation for the ISAP symptom scales

**Figure 1:**
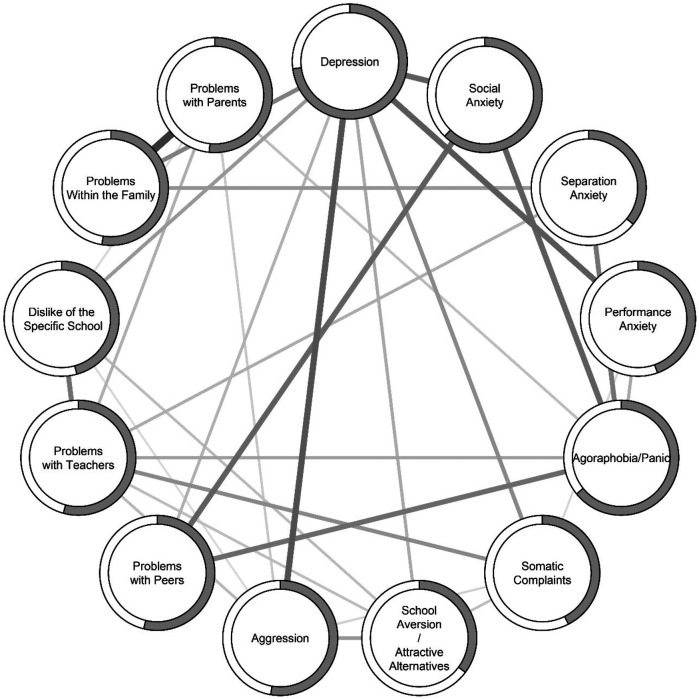
Network model of the 13 ISAP-S scales. Solid lines indicate positive partial correlations between nodes (circles). The shaded border around nodes indicate predictability (*R^2^*) of that node. Average absolute non-zero partial correlation was *r* = .15, strongest partial correlation between “problems within the family” and “problems with parents” (*r* = .37), and weakest partial correlation between “aggression” and “dislike of the specific school” (*r* = .05).

We estimated a network structure based on the 13 sum score variables (nodes) for the symptom scales, yielding partial correlations (edges) between the nodes. All reported correlations are partial correlations. There were 31 edges with non-zero weight. (See also Table 2 for partial correlation coefficients.) Some nodes were more connected than others, as can be seen in Figure 1. The redundancy analysis revealed several overlapping nodes. For the symptoms network, Problems with Teachers and Aggression, Dislike of Specific School and Problems with Teachers, Agoraphobia and Social Anxiety, Problems with Peers and Social Anxiety, and Dislike of Specific School and Aggression shared significant overlap. These overlaps indicate that these combinations of nodes may measure the same underlying construct. However, we chose not to combine the overlapping variables to keep the results comparable with other studies using the ISAP questionnaire. The symptoms network showed high stability, with CS-coefficients of .673 and .593 for edges and strength centrality, respectively. The strongest edge was found between Problems within Family and Problems with Parents, with a partial correlation of .37 (see Table 2). Other strong edges were found for Social Anxiety with Peer Problems (*r* = .24), and with Agoraphobia/Panic (*r* = .25) and Depression (*r* = .25); and for Depression with Performance Anxiety (*r* = .26) and with Aggression (*r* = .28).

**Table 2:** Network edges: partial correlation coefficients for the 13 ISAP-S scale*s.*

Variable	ISAP1	ISAP2	ISAP3	ISAP4	ISAP5	ISAP6	ISAP7	ISAP8	ISAP9	ISAP 10	ISAP 11	ISAP 12
ISAP2	.25											
ISAP3												
ISAP4	.26											
ISAP5		.25	.20	.11								
ISAP6	.16			.05								
ISAP7	.11					.08						
ISAP8	.28					.06	.14					
ISAP9	.10	.24			.22							
ISAP10			.11		.13	.16	.08	.09				
ISAP11	.14		.				.09	.05		.21		
ISAP12	.18		.15									
ISAP13					.09			.07		.10	.05	.37

For ease of interpretation, only non-zero-partial correlations are displayed. Average absolute partial correlation (excluding zero) was 0.15.

ISAP1, depression; ISAP2, social anxiety; ISAP3, separation anxiety; ISAP4, performance anxiety; ISAP5, agoraphobia/panic; ISAP6, somatic complaints; ISAP7, school aversion/attractive alternatives; ISAP8, aggression; ISAP9, problems with peers; ISAP10, problems with teachers; ISAP11, dislike of the specific school; ISAP12, problems within the family; ISAP13, problems with parents.

### 3.3 Centrality estimation for the ISAP symptom scales

**Figure 2:**
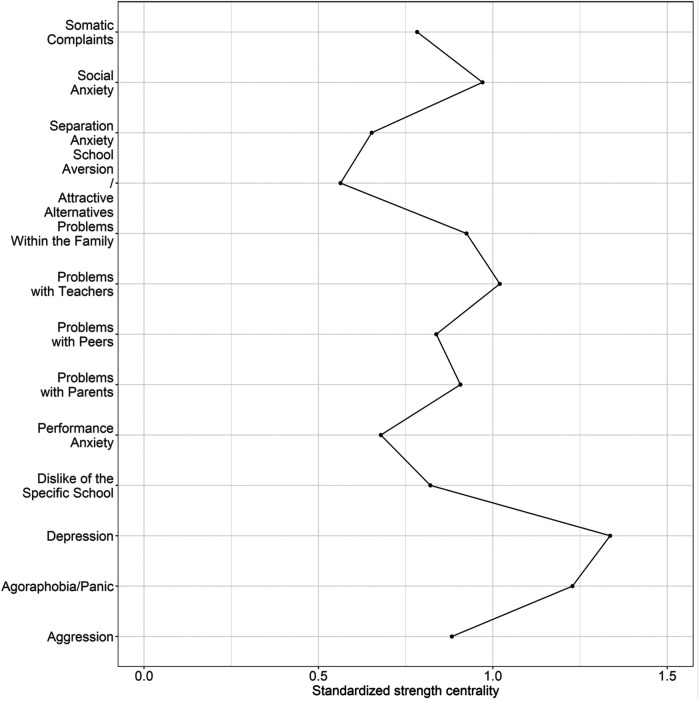
Strength centrality estimates for the 13 ISAP-S scales. Absolute standardized strength centrality scores per node, higher values indicate a higher centrality in the network.

Figure 2 shows the centrality estimate *strength*. The strength measures the sum of all absolute partial correlations that are associated with the edge. The strength is also a measure of centrality in the model. Depression is the most central node according to this measure, while Agoraphobia/Panic was the next most central node in the network, although they did not differ in strength based on the bootstrapped significance test (bootstrapped confidence interval: −0.50, 0.10).

### 3.4 Network estimation for the ISAP function scales

**Figure 3:**
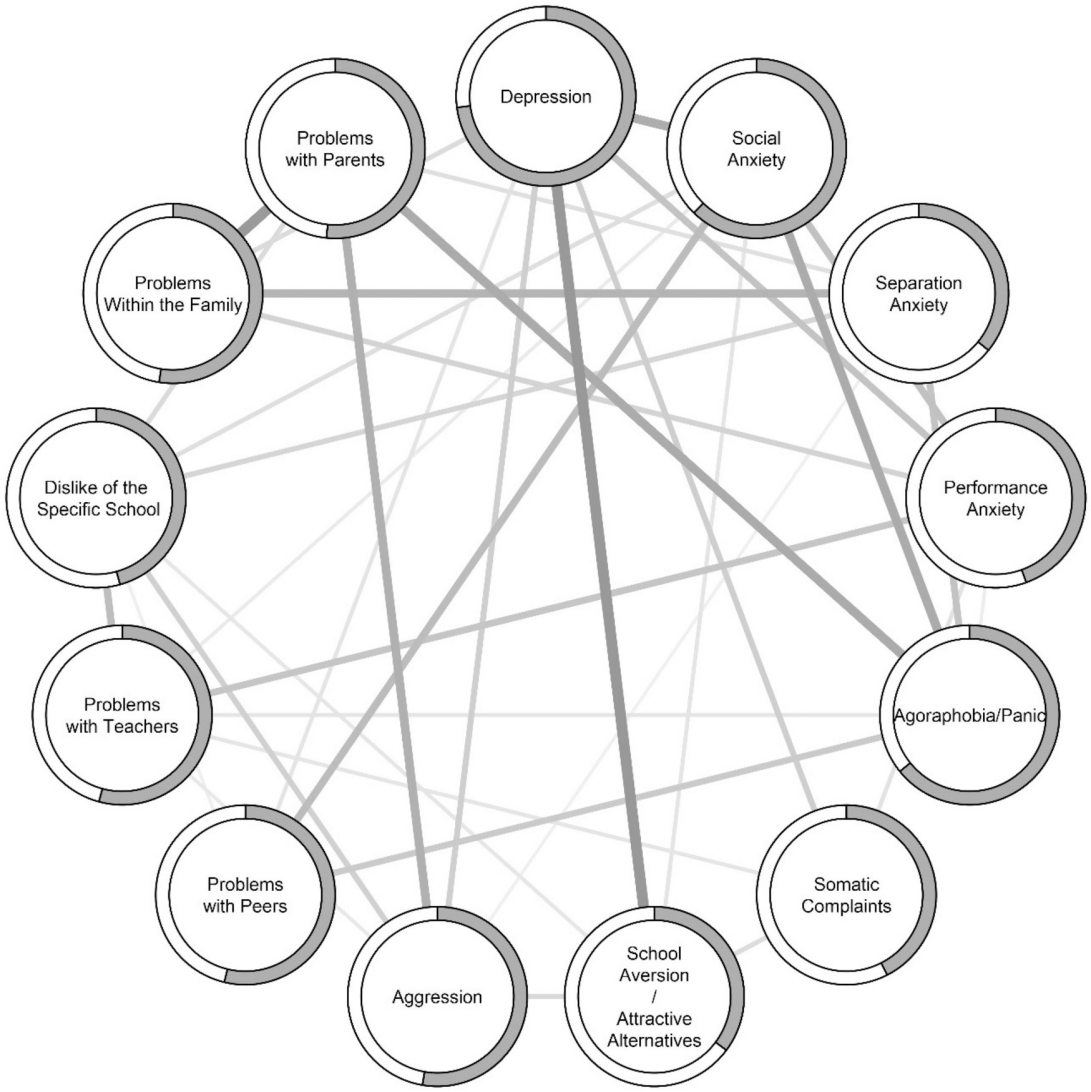
Network model of the 13 ISAP-F scales. Solid lines indicate positive partial correlations between nodes (circles). The shaded border around nodes indicate predictability (*R^2^*) of that node. Average absolute non-zero partial correlation was *r* = .15, strongest partial correlation between “depression” and “school aversion/attractive alternatives” (*r* = .32), and weakest partial correlation between “separation anxiety” and “aggression” (*r* = .05).

When we estimated a network structure based on the 13 sum score variables (nodes) for the function scales, we found a pattern similar to the symptom network, with some small differences. The redundancy analysis revealed several overlapping nodes. In the functions network, Problems with Teachers and Problems with Peers as well as Problems with Peers and Performance Anxiety shared significant overlap. The functions network showed high stability, with CS-coefficients of .593 and .593 for edges and strength centrality, respectively. The strongest correlations (see Table 3) were between Depression and School Aversion (*r* = .32), Depression and Social Anxiety (*r* = .25), Social Anxiety and Agoraphobia/Panic (*r* = .26), and Separation Anxiety and Problems Within the Family (*r* = .23). A strong link, similar to that found in the symptoms network, was observed between Problems with Parents and Problems Within the Family (*r* = .31) (Figure 3).

**Table 3:** Network edges: partial correlation coefficients for the 13 ISAP-F scales.

Variable	ISAP1	ISAP2	ISAP3	ISAP4	ISAP5	ISAP6	ISAP7	ISAP8	ISAP9	ISAP10	ISAP11	ISAP12
ISAP2	.25											
ISAP3												
ISAP4	.18	.19										
ISAP5		.26	.18	.06								
ISAP6	.15			.09								
ISAP7	.32	.08				.11						
ISAP8	.15		.05				.12					
ISAP9	.08	.21			.16							
ISAP10		.06		.18	.08	.08		.07				
ISAP11		.10	.13				.08	.14	.05	.20		
ISAP12	.11		.23	.14								
ISAP13			.10		.26			.25			.11	.31

For ease of interpretation, only non-zero-partial correlations are displayed.

Average absolute partial correlation (excluding zero) was 0.15.

ISAP1, depression; ISAP2, social anxiety; ISAP3, separation anxiety; ISAP4, performance anxiety; ISAP5, agoraphobia/panic; ISAP6, somatic complaints; ISAP7, school aversion/attractive alternatives; ISAP8, aggression; ISAP9, problems with peers; ISAP10, problems with teachers; ISAP11, dislike of the specific school; ISAP12, problems within the family; ISAP13, problems with Parents.

### 3.5 Centrality estimation for the ISAP function scales

**Figure 4:**
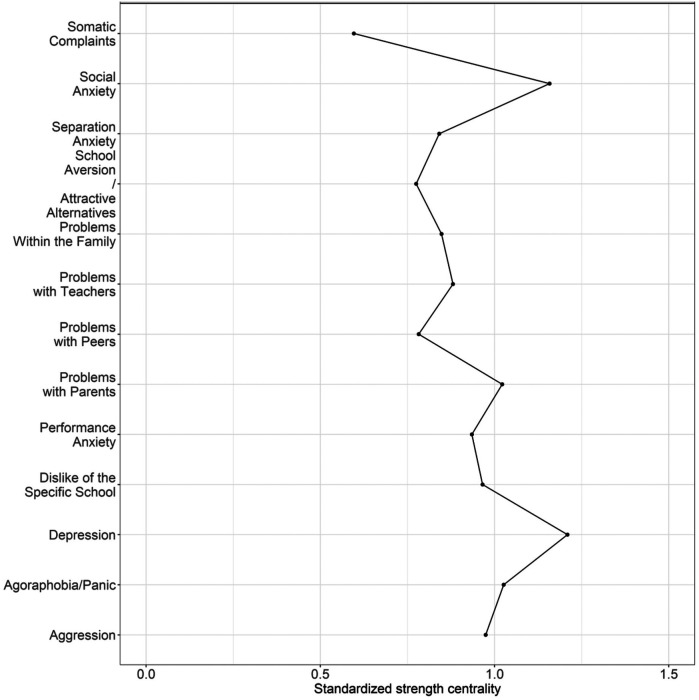
Strength centrality estimates for the 13 ISAP-F scales. Absolute standardized strength centrality scores per node, higher values indicate a higher centrality in the network.

Figure 4 shows the centrality estimate *strength* for the ISAP function scales. Depression is the most central node, according to this measure, and Social Anxiety was the next central node in the network, although they did not differ in strength based on the bootstrapped significance test (bootstrapped confidence interval: −0.57, 0.01).

## 4 Discussion

This study represents an early effort to better understand SAPs utilizing network analysis. The aim was to identify central symptoms associated with absence and the functions served by these symptoms, among adolescents reporting 10% or more school days missed. Specifically, we estimated edges (partial correlations) between nodes (sum scores of variables) for scales derived from the ISAP, namely 13 symptom scales and 13 function scales.

Of the original sample of 1,866 youth, we selected the 349 who reported ≥10% absence for analysis. This proportion (18.7%) underscores the widespread occurrence of absence at levels considered problematic within the studied population. Furthermore, within this subsample, relatively high rates of scale scores ≥1.5 were observed, indicating that a substantial proportion of youth experienced symptoms suggestive of various psychosocial challenges.

### 4.1 The centrality of symptoms associated with SAPs, and the centrality of functions served by these symptoms

Considering the findings from both networks—the network of symptom scales and the network of function scales—depression emerges as an important construct in understanding SAPs. Specifically, Depression was the most central node in both networks. However, although Depression was descriptively the most central symptom, bootstrap difference testing indicated that it was not significantly more central than Agoraphobia/Panic in the symptoms network, or Social Anxiety in the functions network. This pattern suggests that a cluster of symptoms, including Depression, Social Anxiety, and Agoraphobia/Panic, may represent particularly important processes in SAPs.

Prior research on SAPs has highlighted the prominent role of depression (48). Building on this, the present study distinguished between Depression as a symptom and as a function, allowing for a more nuanced examination of the role of Depression in SAPs. Depression as a symptom reflects the young person's experience of depressive feelings and emotional distress within the school-related emotional landscape. In contrast, Depression as a function—or reason for absence—suggests that absence from school may serve to manage depressive symptoms, or that the young person's overall functioning is severely affected by depressive symptomatology.

In the current study, both the symptom and function network analyses revealed that Depression was strongly associated with School Aversion/Attractive Alternatives—a construct resembling truancy (2). The association between Depression and School Aversion was stronger in the function network than in the symptom network, suggesting heightened functional difficulties related to depressive symptomatology. This finding adds nuance to earlier evidence from Finning et al.'s (48) meta-analysis, which showed that depression was associated not only with overall absence but also with specific types of absence, including emotion-related absence and truancy. Moderate-to-large associations were observed between depression and emotion-related absence, whereas small-to-moderate associations were found for unexcused absences typically equated with truancy (48). Consistent with this pattern, a review by Heyne et al. (49) suggested that depression is more characteristic of emotion-related absence than of truancy.

A further pattern observed in the current study was the association between Depression and Aggression, consistent with previous research (50, 51). These findings suggest that some youth with SAPs who ostensibly display aggressive behavior may also experience depressive symptoms that are less readily visible (mixed disorder of conduct and emotions; ICD-10: F92x). Understanding this association is important, because among youth with SAPs—especially when the absence is characteristic of truancy—depressive symptomatology may go unnoticed, being overshadowed by behavioral issues. The redundancy analysis revealed that Aggression overlapped with having Problems with Teachers and Dislike of the Specific School. This pattern suggests that these factors account for similar variance in absence, possibly reflecting a broader pattern of school-related conflict and disengagement. However, results from the confirmatory factor analysis based on the same dataset suggest that they represent distinct latent constructs (32, 33). That is, rather than indicating that they are identical constructs, the overlap highlights how aggressive behavior, strained teacher relationships, and negative attitudes toward school may co-occur and reinforce one another in ways that contribute to problematic absence.

From a network perspective, symptoms may form self-sustaining loops, whereby strong interconnections help maintain them even after the original trigger has subsided (35, 52). However, because our data are cross-sectional, such feedback processes cannot be directly tested and should therefore be considered speculative. Consistent with prior network studies of adolescent depression (53, 54), we found that Depression and specific symptoms such as sadness and loneliness occupied central positions, supporting the view that depressive symptoms often function as structurally central features within broader symptom networks. This centrality may reflect the frequent co-occurrence of depressive symptomatology among adolescents experiencing persistent difficulties or comorbidity (55, 56). Nevertheless, potential causal or sustaining dynamics should be interpreted with caution when based on cross-sectional designs (57).

Agoraphobia/Panic emerged as another central node in the symptoms and functions networks. Prior research has consistently shown associations between anxiety and school absence (58). A meta-analysis by Finning et al. (48) identified social anxiety, separation anxiety, and generalized anxiety as commonly associated with absence, and these forms of anxiety also showed many edges in the current symptoms and functions networks. While Agoraphobia/Panic has received comparatively less attention in the field of school attendance, a study by Hella and Bernstein (59) demonstrated its relevance to absence. In the present analyses, Agoraphobia/Panic overlapped considerably with Social Anxiety in the redundancy analysis, suggesting shared explanatory variance. However, the CFA based on the same dataset suggested that they remain separable constructs (32, 33).

Social Anxiety also emerged as a central node in the functions network. It was linked to Depression, Agoraphobia/Panic, and Problems with Peers—a pattern consistent with prior meta-analytical evidence (48). Studies investigating interventions for emotion-related absence have shown less favorable outcomes when young people experience social anxiety (1). Social anxiety may contribute to strong avoidance of the school environment as a means of evading peer interactions. Ingul and Nordahl (21) similarly reported higher levels of social anxiety and peer problems among non-attending youth in Norway, and problematic social functioning appears to be a distinguishing feature of emotion-related absence in adolescence (60).

### 4.2 Relationships between symptoms, and relationships between functions

Previous studies employing path analytical methods have documented several pathways akin to those that emerged in the current study, such as connections between variables like depression and anxiety (22), separation anxiety and problems with parents (61), and social anxiety and problems with peers (62). The distinctive strength of network analysis lies in its ability to transcend predefined (*a priori*) pathways, estimating all potential pathways and subsequently regularizing them to zero if they prove too weak. This unique feature allows for the identification of pathways that might otherwise be overlooked. Given the relative newness of the ISAP, considering the patterns observed across its symptom and function subscales—although not part of a formal research question—offers valuable insight into how SAP-related processes may interact.

The intricate relationships among symptoms reported by young people displaying SAPs, and among the functions of these symptoms, are reflected in the complexity of interconnectedness between the nodes presented in Figures 1, 3, respectively. The symptoms network reveals 31 significant associations, and the functions network reveals 36 significant associations. This suggests that when adolescents with school absence of 10% or more have high scores on a symptoms and/or functions subscale, they are likely to also experience problems related to another subscale. The effect sizes on most nodes were large, indicating that when accounting for the effect of all factors in the symptoms model simultaneously and all factors in the functions model simultaneously, these factors collectively account for a substantial portion of the variance attributed to the respective node.

wThe strongest edge in both the symptoms network (.37) and the functions network (.31) was between Problems within the Family and Problems with Parents, after controlling for all other nodes. This moderate association is theoretically consistent, given that both constructs refer to challenges situated within the family domain, albeit with slightly different emphases. Problems within the Family contains specific reference to events/specific problems within the family, whereas Problems with Parents includes items about feeling rejected or overlooked by parents. The former node may be thought to include contextual factors, such as parental unemployment, or family dysfunction or conflicts. This could also be reflected in the association of the node with separation anxiety—cases when the anxiety is related to worry about parent wellbeing or home environment stability while the young person is at school. Conversely, Problems with Parents may reflect the quality of the parent-child relationship and parenting style, as evidenced by the emotional responses of the young person. The findings suggest a bidirectional influence between family dynamics and parent-child relationships.

Most associations at both the symptom and function levels were small to moderate in size and largely involved the same nodes across the symptoms and functions networks. Thus, the rationale for the differentiation between symptoms and the function of symptoms needs to be considered. The mean values for the symptoms scales were consistently higher than the mean values for the equivalent functions scales, possibly reflecting the generally high level of functioning in the current sample. That is, the participants may report symptoms, but may not experience functional difficulties related to the symptoms (i.e., report no function of the node). Furthermore, at the individual level, a separate analysis of symptoms and functions is still warranted for treatment planning purposes.

### 4.3 Strengths and limitations of the study

The current study capitalizes on the strengths of network analysis, a robust statistical approach enabling the simultaneous estimation of all potential connections. In this context, we were able to examine connections between symptoms often associated with SAPs, and the connections between the functions those symptoms serve with respect to absence from school. This methodological choice yielded valuable insights, highlighting the central position of depression among symptoms of SAPs and the reasons for absence from school. Additionally, a notable strength of this study is the incorporation of a sizable community sample, enhancing the generalizability of the study's findings. The network showed high stability in our analyses, meaning that it is likely that the networks would replicate in other similar samples.

Our use of the ISAP to measure 48 symptoms potentially associated with SAPs, along with the function each symptom serves in youth' absence, can be considered both a strength and limitation of the study. On the one hand, the ISAP allows for the simultaneous measurement of symptoms and the function of each symptom, surpassing the scope of other questionnaires that focus on either symptoms or functions, but not both. On the other hand, the novelty of the ISAP presents a potential limitation, given the limited—albeit growing –information on its psychometric properties. Overall, we suggest that the strength of using the ISAP outweighs the limitation, as no other available instrument provides information about the function of each specific symptom assessed.

In general, all ISAP constructs had acceptable to excellent internal reliability in the current study. Several subscales of the ISAP were identified as redundant in the networks. Whereas redundancy analysis identified overlap between several factors, the CFA models of ISAP using the same sample (32) and other samples (33) suggested that they represent distinct latent constructs. This difference arises because redundancy analysis is outcome-oriented, highlighting predictors that account for similar variance in the dependent variables, while CFA evaluates the measurement structure of the constructs themselves. Thus, factors can appear redundant in terms of explanatory power without being indistinguishable at the latent construct level.

Six limitations merit consideration. It is important to adopt a cautious approach in interpreting the findings in light of these limitations.

First, the study's reliance on self-reported data introduces potential recall and response biases, as participants' perceptions and memories may not fully capture their actual experiences or behaviors.

Second, the use of a non-random, self-selected sample limits the generalizability of the findings. It is possible that schools already engaged with attendance-related issues were more inclined to participate, resulting in a sample that may not fully represent all school contexts. Nevertheless, within participating schools and classes, most students took part, which helps mitigate—but does not eliminate—these sampling concerns.

Third, youth experiencing more severe SAPs may have been absent on data collection days. Given the limited outreach capacity for this extensively absent group, their underrepresentation introduces the likelihood of non-representative responses.

Fourth, the categorical measurement of “days absent” may have reduced the specificity of subsequent analyses. The ISAP questionnaire's non-intuitive categorical response options may also have been difficult for respondents to interpret.

Fifth, the study did not include some symptoms previously associated with SAPs, such as neuropsychiatric conditions such as autism spectrum disorder (63) and attention deficit hyperactivity disorder (64, 65), or symptoms associated with eating disorders (64).

Sixth, data collection occurred during the latter phase of the COVID-19 pandemic, potentially leading to distinct network structures compared with pre- or post-pandemic contexts. Although schools in Finland had resumed in-person instruction by that time, students' routines, well-being, and perceptions of school were likely still influenced by earlier disruptions caused by school closures and public health measures. These lingering effects may have shaped both the experiences reported by students and the observed network structures, thereby limiting the generalisability of the findings to fully post-pandemic conditions. Nevertheless, all students had returned to at least partial in-person instruction during data collection, marking a period of relative normalisation. The data therefore likely capture enduring effects of the pandemic without being confounded by the acute disruptions of earlier phases. This contextualises the findings and underscores the need for future replication studies in clearly post-pandemic settings.

At the same time, important shifts in school attendance research were already underway prior to the pandemic (77). Specifically, there has been a growing move from focusing primarily on individual and family determinants of absence toward considering broader systemic and structural influences (78), alongside a shift from addressing absence to actively promoting attendance (66). While the pandemic drew increased attention to school attendance issues, it is best understood as having accelerated—rather than initiated—these ongoing developments.

### 4.4 Practical implications and suggestions for future research

Adolescence is a pivotal period for identity development (67) and the safeguarding of mental well-being (68), making it a critical time for identifying and addressing SAPs, which tend to peak during this stage of development (1, 69). This underscores the importance of developmentally sensitive interventions that promote school re-entry, engagement, and well-being (1).

Given that SAPs are prone to become chronic when co-occuring with mental health symptoms (1, 70), timely and focused intervention is essential. Intervention efforts should strategically address symptoms that impede progress toward increased school attendance (1, 71, 72). The findings suggest the importance of ensuring that depressive symptoms are included in the assessment process—even when other mental health challenges initially appear salient. This emphasis does not minimize the relevance of other symptoms but acknowledges depression as a potentially central contributor to SAPs that might otherwise be overlooked. Depending on assessment outcomes, interventions may need to address depressive symptoms alongside social anxiety, school aversion, bullying, and related concerns. Several existing intervention protocols, including @school (73, 74), Back2School (75), and Modular Treatment (72), already include modules targeting depression. Future research could investigate whether prioritising central nodes (e.g., depression, social anxiety) leads to measurable reductions in absence. Combining network analysis with intervention trials may ultimately yield more efficient and personalised treatment strategies.

The results also indicated that symptoms and their functions tend to cluster, suggesting that certain emotional and contextual difficulties commonly co-occur. For example, problems within the family, difficulties in the parent-child relationship, and separation anxiety often appeared together. Recognizing these clusters highlights how interconnected factors may maintain or exacerbate SAPs. This has important implications for both assessment and intervention, as practitioners can target groups of related factors rather than addressing symptoms in isolation. Interventions that strengthen family relationships, improve communication, and reduce anxiety around separation may therefore alleviate multiple, interlinked challenges.

Future research should take into account the research context—whether community-based or specialist-referred. SAPs vary along a continuum of severity (76), and the current study included youth with absences ranging from 5 to over 48 days, most at the lower end. Thus, the findings likely reflect network patterns characteristic of milder forms of SAPs, which may differ from those observed in more severe or clinically referred samples. Investigating whether similar network structures of symptoms and functions emerge in such samples represents a valuable next step.

In addition, future studies should consider the broader context in which data are collected. The present study was conducted during the latter phase COVID-19 pandemic, a period marked by unique disruptions to students' social and school lives. Replicating the current analyses in post-pandemic conditions would help clarify the extent to which the observed patterns reflect enduring features of SAPs vs. context-specific patterns shaped by the pandemic environment.

## 5 Conclusion

This study is one of the first to apply network analysis to better understand SAPs through the examination of a broad range of self-reported symptoms and their functions in relation to school absence. The analysis identified depression as a central node, interconnected with many other symptoms and functions, suggesting that depressive symptoms play a structurally important role in the broader network of SAPs. While causal inferences cannot be drawn from cross-sectional data, the findings indicate that depression may represent a useful target for intervention and prevention efforts, particularly through strategies aimed at enhancing emotion regulation and life skills associated with a reduced risk of mental health problems. Interventions that address depressive symptoms may also yield broader benefits by alleviating co-occurring difficulties among youth at risk of, or already experiencing, SAPs.

## Data Availability

Data is available upon reasonable request from the first author.

## Appendix 1. Item number of variables that loaded on each factor.

**Table:**

Factor number	Item numbers	Factor label
ISAP 1	1, 8, 35, 41, 45, 48	Depression
ISAP 2	9, 12, 24, 32, 47	Social Anxiety
ISAP 3	6, 25, 27, 38	Separation Anxiety
ISAP 4	28, 29, 44	Performance Anxiety
ISAP 5	7, 19, 33, 46	Agoraphobia/Panic
ISAP 6	10, 17, 36	Somatic Complaints
ISAP 7	2, 15, 18, 21	School Aversion/Attractive Alternatives
ISAP 8	4, 16, 37	Aggression
ISAP 9	3, 22, 23, 26	Problems with Peers
ISAP 10	5, 11, 42	Problems with Teachers
ISAP 11	14, 30, 39	Dislike of the Specific School
ISAP 12	31, 34, 43	Problems Within the Family
ISAP 13	13, 20, 40	Problems with Parents

## References

[1] Heyne D. Developmental issues associated with adolescent school refusal and cognitive-behavioral therapy manuals. Z Kinder Jugendpsychiatr Psychother. (2022) 50(6):471–94. 10.1024/1422-4917/a000881 35762908

[2] Heyne D Gren-Landell M Melvin G Gentle-Genitty C. Differentiation between school attendance problems: why and how? Cogn Behav Pract. (2019) 26(1):8–34. 10.1016/j.cbpra.2018.03.006

[3] Kearney CA Graczyk PA. A multidimensional, multi-tiered system of supports model to promote school attendance and address school absenteeism. Clin Child Fam Psychol Rev. (2020) 23(3):316–37. 10.1007/s10567-020-00317-1 32274598

[4] Kearney CA. Managing School Absenteeism at Multiple Tiers: An Evidence-Based and Practical Guide for Professionals. New York: Oxford University Press (2016). 10.1093/med:psych/9780199985296.001.0001

[5] Graczyk PA Gentle-Genitty C Humm Patnode A Moulton SE. Searching for consistency in attendance data recording, reporting, and utilization in the USA. Orbis Scholae. (2023) 16:1–29. 10.14712/23363177.2023.6

[6] Kreitz-Sandberg S Backlund Å Fredriksson U Isaksson J Rasmusson M Gren Landell M. Recording and reporting school attendance and absence: international comparative views on attendance statistics in Sweden, Germany, England, and Japan. Orbis Scholae. (2023) 16:1–26. 10.14712/23363177.2023.9

[7] Maeda N. What does school attendance mean in Japanese compulsory education schools? Analysing the national annual report. Orbis Scholae. (2023) 16:1–13. 10.14712/23363177.2023.4

[8] Skedgell K Kearney CA. Predictors of school absenteeism severity at multiple levels: a classification and regression tree analysis. Child Youth Serv Rev. (2018) 86:236–45. 10.1016/j.childyouth.2018.01.043

[9] Egger HL Costello EJ Angold A. School refusal and psychiatric disorders: a community study. J Am Acad Child Adolesc Psychiatry. (2003) 42(7):797–807. 10.1097/01.CHI.0000046865.56865.79 12819439

[10] Havik T Bru E Ertesvåg SK. Assessing reasons for school non-attendance. Scand J Educ Res. (2015) 59(3):316–36. 10.1080/00313831.2014.904424

[11] Heyne D King NJ. Treatment of school refusal. In: Barrett PM Ollendick TH, editors. Handbook of Interventions Thatwork with Children and Adolescents: Prevention and Treatment. Chichester, England: John Wiley (2004). p. 243–72.

[12] Alanko K Melander K Ranta K Engblom J Kosola S. Time trends in adolescent school absences and associated bullying involvement between 2000 and 2019: a nationwide study [Preprint]. (2023). 10.21203/rs.3.rs-2651595/v1 PMC12095371 37632555

[13] Havik T Ingul JM. How to understand school refusal. Front Educ. (2021) 6:715177. Available online at: https://www.frontiersin.org/articles/10.3389/feduc.2021.715177.

[14] Ricking H Schulze GC. Research and management of school absenteeism in Germany: educational perspectives. UrbanScope. (2019) 10:39–54.

[15] Alanko K Söderberg P Lagerström M Laakso M-J Junttila N. The association between social outsiderhood and school absence is mediated by internalizing symptoms. School Ment Health. (2025) 17:1041–53. 10.1007/s12310-025-09793-8

[16] Pijl EK Vanneste YTM de Rijk AE Feron FJM Mathijssen J. The prevalence of sickness absence among primary school pupils—reason to be worried? BMC Public Health. (2021) 21(1):170. 10.1186/s12889-021-10193-1 33472603 PMC7816510

[17] Dee TS. Higher chronic absenteeism threatens academic recovery from the COVID-19 pandemic. Proc Natl Acad Sci USA (2024) 121(3):e2312249121. 10.1073/pnas.2312249121 38194454 PMC10801904

[18] Wolf K Schmitz J. Scoping review: longitudinal effects of the COVID-19 pandemic on child and adolescent mental health. Eur Child Adolesc Psychiatry. (2024) 33(5):1257–312. 10.1007/s00787-023-02206-8 37081139 PMC10119016

[19] Gonzálvez C Kearney CA Vicent M Sanmartín R. Assessing school attendance problems: a critical systematic review of questionnaires. Int J Educ Res. (2021) 105:101702. 10.1016/j.ijer.2020.101702

[20] Melvin GA Heyne D Gray KM Hastings RP Totsika V Tonge BJ The Kids and Teens at School (KiTeS) framework: an inclusive bioecological systems approach to understanding school absenteeism and school attendance problems. Front Educ. (2019) 4:61. 10.3389/feduc.2019.00061

[21] Ingul JM Nordahl HM. Anxiety as a risk factor for school absenteeism: what differentiates anxious school attenders from non-attenders? Ann Gen Psychiatry. (2013) 12(1):25–33. 10.1186/1744-859X-12-25 23886245 PMC3726429

[22] Cummings CM Caporino NE Kendall PC. Comorbidity of anxiety and depression in children and adolescents: 20 years after. Psychol Bull. (2014) 140:816–45. 10.1037/a0034733 24219155 PMC4006306

[23] Baglioni C Battagliese G Feige B Spiegelhalder K Nissen C Voderholzer U Insomnia as a predictor of depression: a meta-analytic evaluation of longitudinal epidemiological studies. J Affect Disord. (2011) 135(1–3):10–9. 10.1016/j.jad.2011.01.011 21300408

[24] Crouse JJ Carpenter JS Song YJC Hockey SJ Naismith SL Grunstein RR Circadian rhythm sleep–wake disturbances and depression in young people: implications for prevention and early intervention. Lancet Psychiatry. (2021) 8(9):813–23. 10.1016/S2215-0366(21)00034-1 34419186

[25] Donnelly HK Richardson D Solberg SV. Understanding somatic symptoms associated with South Korean adolescent suicidal ideation, depression, and social anxiety. Behav Sci. (2021) 11(11):151. 10.3390/bs11110151 34821612 PMC8615240

[26] Bringmann LF Lemmens LHJM Huibers MJH Borsboom D Tuerlinckx F. Revealing the dynamic network structure of the beck depression inventory-II. Psychol Med. (2015) 45(4):747–57. 10.1017/S0033291714001809 25191855

[27] Kearney CA Albano AM. The functional profiles of school refusal behavior. Diagnostic aspects. Behav Modif. (2004) 28(1):147–61. 10.1177/0145445503259263 14710711

[28] Kearney CA Silverman WK. A preliminary analysis of a functional model of assessment and treatment for school refusal behavior. Behav Modif. (1990) 14(3):340–66. 10.1177/01454455900143007 2375736

[29] Kearney C. Identifying the function of school refusal behavior: a revision of the school refusal assessment scale. J Psychopathol Behav Assess. (2002) 24(4):235–45. 10.1023/A:1020774932043

[30] Knollmann M Reissner V Hebebrand J. Towards a comprehensive assessment of school absenteeism: development and initial validation of the inventory of school attendance problems. Eur Child Adolesc Psychiatry. (2019) 28(3):399–414. 10.1007/s00787-018-1204-2 30043236

[31] Kearney CA. Forms and functions of school refusal behavior in youth: an empirical analysis of absenteeism severity. J Child Psychol Psychiatry. (2007) 48(1):53–61. 10.1111/j.1469-7610.2006.01634.x 17244270

[32] Alanko K Lagerström M. Network analysis of factors related to school attendance problems. [Conference presentation]. Third International Network for School Attendance (INSA) Conference, Egmond aan Zee, Netherlands (2022, October 5–7).

[33] Strömbeck J Heyne D Ferrer-Wreder L Alanko K. Reliability and validity of the Swedish version of the inventory of school attendance problems (ISAP). Eur Child Adolesc Psychiatry. (2025) 34:2069–82. 10.1007/s00787-024-02618-0 39607485 PMC12334511

[34] Borsboom D Cramer AOJ. Network analysis: an integrative approach to the structure of psychopathology. Annu Rev Clin Psychol. (2013) 9:91–121. 10.1146/annurev-clinpsy-050212-185608 23537483

[35] Bansal PS Goh PK Lee CA Martel MM. Conceptualizing callous-unemotional traits in preschool through confirmatory factor and network analysis. J Abnorm Child Psychol. (2020) 48(4):539–50. 10.1007/s10802-019-00611-9 31900834

[36] Epskamp S Borsboom D Fried EI. Estimating psychological networks and their accuracy: a tutorial paper. Behav Res Methods. (2018) 50(1):195–212. 10.3758/s13428-017-0862-1 28342071 PMC5809547

[37] Buuren SV Groothuis-Oudshoorn K. **mice**: multivariate imputation by chained equations in *R*. J Stat Softw. (2011) 45:3. 10.18637/jss.v045.i03

[38] Kowarik A Templ M. Imputation with the R package VIM. J Stat Softw. (2016) 74:1–16. 10.18637/jss.v074.i07

[39] Epskamp S Cramer AOJ Waldorp LJ Schmittmann VD Borsboom D. Qgraph: network visualizations of relationships in psychometric data. J Stat Softw. (2012) 48:1–18. 10.18637/jss.v048.i04

[40] Haslbeck JMB Waldorp LJ. Mgm: estimating time-varying mixed graphical models in high-dimensional data. J Stat Softw. (2020) 93:1–46. 10.18637/jss.v093.i08

[41] Wickham H Averick M Bryan J Chang W McGowan LD François R Welcome to the Tidyverse. J Open Source Softw. (2019) 4(43):1686. 10.21105/joss.01686

[42] Foygel R Drton M. Extended Bayesian information criteria for Gaussian graphical models. arXiv. arXiv:1011.6640 (2010). 10.48550/arXiv.1011.6640

[43] Haslbeck JMB Waldorp LJ. How well do network models predict observations? On the importance of predictability in network models. Behav Res Methods. (2018) 50(2):853–61. 10.3758/s13428-017-0910-x 28718088 PMC5880858

[44] Valente TW. Network interventions. Science. (2012) 337(6090):49–53. 10.1126/science.1217330 22767921

[45] Bringmann LF Elmer T Epskamp S Krause RW Schoch D Wichers M What do centrality measures measure in psychological networks? J Abnorm Psychol. (2019) 128(8):892–903. 10.1037/abn0000446 31318245

[46] Robinaugh DJ Millner AJ McNally RJ. Identifying highly influential nodes in the complicated grief network. J Abnorm Psychol. (2016) 125(6):747–57. 10.1037/abn0000181 27505622 PMC5060093

[47] Jones P. networktools: Tools for Identifying Important Nodes in Networks (p. 1.6.0) [Dataset]. (2017). Available online at: 10.32614/CRAN.package.networktools (Accessed April 1, 2025).

[48] Finning K Ukoumunne OC Ford T Danielson-Waters E Shaw L Jager IRD Review: the association between anxiety and poor attendance at school—a systematic review. Child Adolesc Ment Health. (2019) 24:205–16. 10.1111/camh.12322 32677217

[49] Heyne D Sauter FM Maynard BM. Moderators and mediators of treatments for youth with school refusal or truancy. In: Maric M Prins PJM Ollendick TH, editors. Moderators and Mediators of Youth Treatment Outcomes, online ed. New York: Oxford Academic (2015). p. 230–66. Available online at: 10.1093/med:psych/9780199360345.003.0010 (Accessed 27 October 2025).

[50] Dugré JR Dumais A Dellazizzo L Potvin S. Developmental joint trajectories of anxiety-depressive trait and trait-aggression: implications for co-occurrence of internalizing and externalizing problems. Psychol Med. (2020) 50(8):1338–47. 10.1017/S0033291719001272 31172895

[51] Fanti KA. Understanding heterogeneity in conduct disorder: a review of psychophysiological studies. Neurosci Biobehav Rev. (2018) 91:4–20. 10.1016/j.neubiorev.2016.09.022 27693700

[52] Hevey D. Network analysis: a brief overview and tutorial. Health Psychol Behav Med. (2018) 6(1):301–28. 10.1080/21642850.2018.1521283 34040834 PMC8114409

[53] Mullarkey MC Marchetti I Beevers CG. Using network analysis to identify central symptoms of adolescent depression. J Clin Child Adolesc Psychol. (2019) 48(4):656–68. 10.1080/15374416.2018.1437735 29533089 PMC6535368

[54] Gijzen MWM Rasing SPA Creemers DHM Smit F Engels RCME De Beurs D. Suicide ideation as a symptom of adolescent depression. A network analysis. J Affect Disord. (2021) 278:68–77. 10.1016/j.jad.2020.09.029 32956963

[55] Essau CA de la Torre-Luque A. Comorbidity between internalising and externalising disorders among adolescents: symptom connectivity features and psychosocial outcome. Child Psychiatry Hum Dev. (2023) 54(2):493–507. 10.1007/s10578-021-01264-w 34655358 PMC9977855

[56] Konac D Young KS Lau J Barker ED. Comorbidity between depression and anxiety in adolescents: bridge symptoms and relevance of risk and protective factors. J Psychopathol Behav Assess. (2021) 43(3):583–96. 10.1007/s10862-021-09880-5 34720388 PMC8550210

[57] Spiller TR Levi O Neria Y Suarez-Jimenez B Bar-Haim Y Lazarov A. On the validity of the centrality hypothesis in cross-sectional between-subject networks of psychopathology. BMC Med. (2020) 18:297. 10.1186/s12916-020-01740-5 33040734 PMC7549218

[58] Heyne DA Kearney CA Finning K. Mental health and attendance at school: setting the scene. In: Finning K Ford T Moore D, editors. Mental Health and Attendance at School. Cambridge: Cambridge University Press (2022). p. 1–21. 10.1017/9781911623151.002

[59] Hella B Bernstein GA. Panic disorder and school refusal. Child Adolesc Psychiatr Clin. (2012) 21(3):593–606. 10.1016/j.chc.2012.05.012 22800996

[60] Carpentieri R Iannoni ME Curto M Biagiarelli M Listanti G Andraos MP School refusal behavior: role of personality styles, social functioning, and psychiatric symptoms in a sample of adolescent help-seekers. Clin Neuropsychiatry. (2022) 19(1):20–8. 10.36131/cnfioritieditore20220104 35360464 PMC8951164

[61] Dabkowska M Araszkiewicz A Dabkowska A Wilkosc M Dabkowska M Araszkiewicz A Separation anxiety in children and adolescents. In: Selek S, editor. Different Views of Anxiety Disorders. London: IntechOpen (2011). p. 313–38. 10.5772/22672

[62] Flanagan KS Erath SA Bierman KL. Unique associations between peer relations and social anxiety in early adolescence. J Clin Child Adolesc Psychol. (2008) 37(4):759–69. 10.1080/15374410802359700 18991127

[63] Munkhaugen EK Gjevik E Pripp AH Sponheim E Diseth TH. School refusal behaviour: are children and adolescents with autism spectrum disorder at a higher risk? Res Autism Spectr Disord. (2017) 41–42:31–8. 10.1016/j.rasd.2017.07.001

[64] John A Friedmann Y DelPozo-Banos M Frizzati A Ford T Thapar A. Association of school absence and exclusion with recorded neurodevelopmental disorders, mental disorders, or self-harm: a nationwide, retrospective, electronic cohort study of children and young people in Wales, UK. Lancet Psychiatry. (2022) 9(1):23–34. 10.1016/S2215-0366(21)00367-9 34826393 PMC8674147

[65] Niemi S Lagerström M Alanko K. School attendance problems in adolescent with attention deficit hyperactivity disorder. Front Psychol. (2022) 13:1017619. 10.3389/fpsyg.2022.1017619 36506967 PMC9726763

[66] Heyne D Gentle-Genitty C Melvin GA Keppens G O’Toole C McKay-Brown L. Embracing change: from recalibration to radical overhaul for the field of school attendance. Front Educ. (2024) 8:1251223. 10.3389/feduc.2023.1251223

[67] Dahl RE. Adolescent brain development: a period of vulnerabilities and opportunities. Keynote address. Ann N Y Acad Sci. (2004) 1021(1):1–22. 10.1196/annals.1308.001 15251869

[68] Neff KD McGehee P. Self-compassion and psychological resilience among adolescents and young adults. Self Identity. (2010) 9(3):225–40. 10.1080/15298860902979307

[69] Vaughn MG Maynard BR Salas-Wright CP Perron BE Abdon A. Prevalence and correlates of truancy in the US: results from a national sample. J Adolesc. (2013) 36(4):767–76. 10.1016/j.adolescence.2013.03.015 23623005 PMC3713173

[70] Knollmann M Knoll S Reissner V Metzelaars J Hebebrand J. School avoidance from the point of view of child and adolescent psychiatry: symptomatology, development, course, and treatment. Dtsch Arztebl Int. (2010) 107(4):43–9. 10.3238/arztebl.2010.0043 20165699 PMC2822958

[71] Heyne D Brouwer-Borghuis M. Signposts for school refusal interventions, based on the views of stakeholders. Contin Educ. (2022) 3(1):25. 10.5334/cie.42 38774290 PMC11104337

[72] Reissner V Jost D Krahn U Knollmann M Weschenfelder A-K Neumann A The treatment of school avoidance in children and adolescents with psychiatric illness. Dtsch Arztebl Int. (2015) 112(39):655–62. 10.3238/arztebl.2015.0655 26479485 PMC4627210

[73] Heyne D Sauter FM van Widenfelt BM Vermeiren R Westenberg PM. School refusal and anxiety in adolescence: non-randomized trial of a developmentally-sensitive cognitive behavioral therapy. J Anxiety Disord. (2011) 25:870–8. 10.1016/j.janxdis.2011.04.006 21602027

[74] Karel E Defourny C Keppens G Graczyk PA Sauter F Heyne D. School-based support for emotion-related attendance challenges: effectiveness of @school when implemented with neurodiverse adolescents, their parents, and school staff. Front Psychol. (2025) 16:1613712. 10.3389/fpsyg.2025.1613712 40625434 PMC12230860

[75] Thastum M Johnsen DB Silverman WK Jeppesen P Heyne DA Lomholt JJ. The Back2School modular cognitive behavioral intervention for youths with problematic school absenteeism: study protocol for a randomized controlled trial. Trials. (2019) 20(1):29. 10.1186/s13063-018-3124-3 30621787 PMC6325742

[76] Kearney CA Gonzálvez C. Unlearning school attendance and its problems: moving from historical categories to postmodern dimensions. Front Educ. (2022) 7:977672. 10.3389/feduc.2022.977672

[77] Kearney CA Childs J. Improving school attendance data and defining problematic and chronic school absenteeism: the next stage for educational policies and health-based practices. Prev Sch Fail. (2022) 67(4):265–75. 10.1080/1045988X.2022.2124222

[78] Kearney CA. Integrating systemic and analytic approaches to school attendance problems: synergistic frameworks for research and policy directions. Child Youth Care F. (2021) 50:701–42. 10.1007/s10566-020-09591-0

